# Phase 1 study of efatutazone, a novel oral peroxisome proliferator-activated receptor gamma agonist, in combination with FOLFIRI as second-line therapy in patients with metastatic colorectal cancer

**DOI:** 10.1007/s10637-013-0056-3

**Published:** 2013-12-15

**Authors:** Yoshito Komatsu, Takayuki Yoshino, Kentaro Yamazaki, Satoshi Yuki, Nozomu Machida, Takahide Sasaki, Ichinosuke Hyodo, Yutaka Yachi, Hiroshi Onuma, Atsushi Ohtsu

**Affiliations:** 1 Hokkaido University Hospital Cancer Center, Kita 14 Nishi 5, Kita-ku, Sapporo, Hokkaido 060-8648 Japan; 2 Department of Gastroenterology and Gastrointestinal Oncology, National Cancer Center Hospital East, Kashiwa, Japan; 3 Division of Gastrointestinal Oncology, Shizuoka Cancer Center, Shizuoka, Japan; 4 Department of Gastroenterology and Hepatology, Hokkaido University Hospital, Sapporo, Japan; 5 Division of Gastroenterology, Graduate School of Comprehensive Human Sciences, University of Tsukuba, Ibaraki, Japan; 6 Clinical Developmental Department II, Daiichi Sankyo Co., Ltd., Tokyo, Japan; 7 Exploratory Oncology Research & Clinical Trial Center, National Cancer Center Hospital East, Kashiwa, Japan

**Keywords:** Efatutazone, Peroxisome proliferator-activated receptor gamma, FOLFIRI, Colorectal cancer

## Abstract

*Background* Efatutazone, a novel oral highly-selective peroxisome proliferator-activated receptor gamma (PPARγ) agonist, has demonstrated some inhibitory effects on disease stabilization in patients with metastatic colorectal cancer (mCRC) enrolled in previous phase I studies. Here, we evaluate the safety and pharmacokinetics of efatutazone combined with FOLFIRI (5-fluorouracil, levo-leucovorin, and irinotecan) as second-line chemotherapy in Japanese patients with mCRC. *Methods* Dose-limiting toxicities (DLTs) were evaluated at 2 efatutazone dose levels of 0.25 and 0.50 mg (the recommended dose [RD] of efatutazone monotherapy) twice daily in combination with FOLFIRI in a 3–9 patient cohort. Furthermore, tolerability at the RD level was assessed in additional patients, up to 12 in total. Blood samples for pharmacokinetics and biomarkers and tumor samples for archival tissues were collected from all patients. *Results* Fifteen patients (0.25 mg, 3; 0.5 mg, 12) were enrolled. No DLTs were observed. Most patients experienced weight increase (100 %) and edema (80.0 %), which were manageable with diuretics. Common grade 3/4 toxicities were neutropenia (93.3 %), leukopenia (46.7 %), and anemia (33.3 %). Stable disease was observed in 8 of the 14 patients evaluable for tumor response. The plasma adiponectin levels increased over time and increased dose. No clear relationship was detected between treatment efficacies and plasma levels of adiponectin as well as the expression levels of PPARγ and the retinoid X receptor in tumor tissues. *Conclusions* Efatutazone combined with FOLFIRI demonstrates an acceptable safety profile and evidence of disease stabilization in Japanese patients with mCRC. The RD for efatutazone monotherapy can be used in combination with FOLFIRI.

## Introduction

Peroxisome proliferator-activated receptor gamma (PPARγ) is a member of the nuclear hormone receptor superfamily. It is activated through its ligands and is involved in the regulation of inflammation, cell cycle progression, cell proliferation, apoptosis, carcinogenesis, and angiogenesis [1–4]. Preclinical studies demonstrated synergistic or additive effects of PPARγ ligands with chemotherapeutic agents on cancer cell apoptosis and growth inhibition, and suggested their potential clinical use in cancer therapy [4–7].

Efatutazone, a novel oral highly-selective thiazolidinedione PPARγ agonist, shows greater potency than second-generation thiazolidinediones such as pioglitazone [8]. In preclinical tumor models, proliferation of human anaplastic thyroid carcinoma and pancreatic tumor cell lines were inhibited *in vitro*, and human colorectal cancer (CRC) and anaplastic thyroid carcinoma cell xenografts were inhibited in nude rodents [9, 10]. PPARγ activity-related adiponectin is also considered a useful biomarker of carcinogenesis and progression of colorectal adenoma [11].

A US Phase 1 study of efatutazone monotherapy in patients with advanced solid malignancies demonstrated acceptable safety and evidence of antitumor activity [12]. A similar study in Japanese patients with metastatic solid tumors confirmed the results (presented in a poster session at the 36^th^ Congress of the European Society for Medical Oncology, 2011) that efatutazone is potentially effective against CRC, by achieving sustained disease stabilization.

In the clinical setting, FOLFOX (5-fluorouracil, levo-leucovorin, and oxaliplatin), CapeOX (capecitabine and oxaliplatin) and FOLFIRI (5-fluorouracil, levo-leucovorin, and irinotecan) regimens are global standard therapies for CRC, demonstrating efficacy and tolerability, and are often chosen as first- and second-line therapies, respectively [13, 14]. The combination of PPARγ ligands with existing chemotherapy is reported to be beneficial for cancer prevention and therapy [4–7], and a novel combination with greater clinical efficacy against advanced refractory tumors including metastatic CRC (mCRC) is needed.

This Phase 1 study was designed to evaluate the safety profile and pharmacokinetics of efatutazone in combination with FOLFIRI as second-line therapy in Japanese patients with mCRC. Secondary objectives included assessment of the preliminary antitumor efficacy of efatutazone, assessment of potential biomarkers of efatutazone including plasma adiponectin, and to determine the recommended dose (RD) of efatutazone in combination with FOLFIRI.

## Materials and methods

### Study design

This Phase 1, open-label, dose-escalation study was conducted at 3 medical centers in Japan between September 2010 and June 2012 (JapicCTI-101230; Clinical Trials Information/JapicCTI, http://www.clinicaltrials.jp/user/cteSearch_e.jsp). It was performed in accordance with the International Conference on Harmonisation Good Clinical Practice Guidelines, the principle of the Declaration of Helsinki and all applicable laws and regulations in Japan. The protocol was reviewed and approved by the Institutional Review Boards of all participating study sites. All patients provided written informed consent before enrollment.

The dose-escalation study had a 3 + 3 design and 2 steps. Since previous Phase 1 studies had indicated an RD of 0.50 mg twice daily (BID) for 4 weeks, we assessed 2 doses of efatutazone, namely, 0.25 and 0.50 mg BID for 4 weeks (1 cycle) in combination with FOLFIRI (irinotecan 180 mg/m^2^ intravenous [IV] infusion over 90 min or longer, levo-leucovorin 200 mg/m^2^ IV infusion over 120 min, and 5-fluorouracil IV continuous infusion 2400 mg/m^2^ over 46 h) once every 2 weeks, in 3 to 9 patients each in Step 1 (dose-escalation phase for evaluation of dose-limiting toxicity [DLT]).

DLTs were defined as follows: (a) grade 3 or higher neutropenia complicated by fever ≥ 38.5 °C or infection, or grade 4 neutropenia with a duration of 7 days or longer; (b) grade 4 thrombocytopenia, or grade 3 thrombocytopenia requiring transfusion; (c) grade 4 anemia; (d) grade 3 or higher pleural or pericardial effusion, peripheral edema or ascites unresponsive to treatment; (e) uncontrollable grade 3 or higher severe fatigue, anorexia, nausea, vomiting or diarrhea, despite maximal supportive therapy; (f) any other grade 3 or higher toxicities than those described in definitions (d) and (e), except for fever without neutropenia and transient electrolyte abnormality.

According to the protocol, if 1 of the first 3 patients at a dose level would experience a DLT, 3 more patients would be enrolled. In case of no DLTs in 3 patients or a maximum of 1 DLT in 6 patients, the study would proceed to the next dose level. In case of 2 DLTs in 6 patients, another 3 patients would be enrolled. RD was based on the Step 1 safety data, and would be administered in combination with FOLFIRI to up to 9 additional patients in Step 2. Administration of efatutazone and FOLFIRI would continue until disease progression or unacceptable toxicity was observed, and then efatutazone and FOLFIRI doses would be reduced based on set guidelines.

### Study population

Inclusion criteria were: age ≥ 20 years; histologically/cytologically confirmed mCRC after failure of first-line chemotherapy; an Eastern Cooperative Oncology Group (ECOG) performance status score of 0 or 1; a measurable lesion; life expectancy of at least 3 months; adequate organ and bone marrow functions documented within 7 days before enrollment, and no blood transfusions within 1 month before enrollment; resolution of toxic effects of prior therapy (except alopecia) to grade 0 or 1 according to the National Cancer Institute Common Terminology Criteria for Adverse Events (NCI-CTCAE), Version 3.0.

Exclusion criteria were: previous irinotecan-based first-line chemotherapy and preexisting severe fluid retention including (edema, pleural effusion and ascites).

### Safety assessment

Safety was assessed at each study visit (every week for 2 cycles, then every 2 weeks) by monitoring adverse events (AEs) and clinical laboratory evaluations. Patients were evaluated for efatutazone-related DLTs in Step 1 (Cycle 1). AEs and laboratory test results were graded according to NCI-CTCAE, Version 3.0.

### Efficacy assessment

Computed tomography or magnetic resonance imaging was performed every 2 cycles for efficacy assessment.

Tumor response was evaluated according to the Response Evaluation Criteria in Solid Tumors, Version 1.1. Overall response rate (ORR) and disease control rate (DCR) were calculated. Progression-free survival (PFS) was defined as time from enrollment to the detection of progressive disease (PD) or death (or the date of last tumor evaluation in stable patients).

### Pharmacokinetics

Blood samples were collected on Day 1 (Cycles 1 and 2) at the following time points: predose (immediately before the morning dose of efatutazone) and postdose (0.5, 1, 2, 3, 4, 6 and 8 to 10 h). Additional samples were collected predose on Days 8 and 15 (Cycle 1). Validated liquid chromatography-tandem mass spectrometry was used to measure the plasma concentration of the free form of efatutazone. The plasma concentration of an active metabolite of irinotecan (SN-38) was also measured immediately after irinotecan hydrochloride (CPT-11) administration in the morning on Day 1 (Cycles 1 and 2).

### Biomarkers

Blood samples for the measurement of plasma adiponectin were collected predose on Days 1, 8 and 15 in Cycle 1, and Day 1 in Cycles 2, 3 and 4. Plasma adiponectin concentrations were determined by quantitative sandwich enzyme immunoassay kit (Quantikine®, R&D systems, Inc., Minneapolis, MN, the USA).

Expression levels of PPARγ and retinoid X receptor (RXR) in archived tumor specimens were studied by immunohistochemistry using PPARγ (C26H12) Rabbit mAb (Cell Signaling Technology, Inc., Danvers, MA, the USA) and Rabbit Anti-Human Retinoic X Receptor Gamma Polyclonal Antibody (Spring Bioscience, Inc., Pleasanton, CA, the USA), respectively.

## Results

### Patient demographics

A total of 15 patients were enrolled and received efatutazone treatment in combination with FOLFIRI. In Step 1, 2 cohorts with 3 patients each received efatutazone at 0.25 and 0.50 mg BID, respectively. In Step 2, an additional 9 patients received efatutazone 0.50 mg BID.

Enrollment started in September 2010, and 15 patients were enrolled by March 2012. Baseline patient characteristics are summarized in Table 1. UGT1A1 genotypes, included 1 homozygous *6/*6 genotype, and 1 homozygous *28/*28 genotype.

**Table 1:** Baseline characteristics (Safety analysis set)

Characteristic	Treatment cohort		
	0.25 mg BID	0.50 mg BID	Overall
	(*n* = 3)	(*n* = 12)	(*n* = 15)
Median age in years	63	64	63
(range)	(56–64)	(41–73)	(41–73)
Gender			
Male	1 (33.3)	6 (50.0)	7 (46.7)
Female	2 (66.7)	6 (50.0)	8 (53.3)
ECOG performance status			
0	2 (66.7)	8 (66.7)	10 (66.7)
1	1 (33.3)	4 (33.3)	5 (33.3)
Primary site			
Rectum	3 (100.0)	7 (58.3)	10 (66.7)
Colon	0	4 (33.3)	4 (26.7)
Colon and rectum	0	1 (8.3)	1 (6.7)
Histological type			
Well differentiated	2 (66.7)	4 (33.3)	6 (40.0)
Moderately differentiated	1 (33.3)	6 (50.0)	7 (46.7)
Poorly differentiated	0	1 (8.3)	1 (6.7)
Others	0	1 (8.3)	1 (6.7)
Previous chemotherapy			
Oxaliplatin-based regimen	2 (66.7)	1 (8.3)	3 (20.0)
Oxaliplatin-based regimen + bevacizumab	0	11 (91.7)	11 (73.3)
Capecitabine monotherapy	1 (33.3)	0	1 (6.7)
UGT1A1 genotype			
Wild (*1/*1)	1 (33.3)	7 (58.3)	8 (66.7)
Heterozygous (*1/*28, *1/*6)	1 (33.3)	4 (33.3)	5 (33.3)
Homozygous (*28/*28, *6/*6, *28/*6)	1 (33.3)^a^	1 (8.3)^b^	2 (13.3)

Values represent the number (%) of subjects

*BID* twice daily, *ECOG* Eastern Cooperative Oncology Group, *n* number of subjects

^a^One subject had UGT1A1 *6/*6 genotype

^b^One subject had UGT1A1 *28/*28 genotype

### Safety

Overall, efatutazone showed acceptable safety at both 0.25 and 0.50 mg BID. No DLTs were observed during the evaluation period (Cycle 1, Step 1). The median duration (range) of efatutazone treatment was 152.0 (71–157) days in the 0.25 mg BID group, 62.5 (21–241) days in the 0.50 mg BID group, and 67.0 (21–241) days in the overall study population.

Treatment-emergent AEs (TEAEs) that occurred in 3 or more patients throughout the study period are summarized in Table 2. Most patients experienced weight increase (100 %) and edema (80.0 %), usually at a severity of ≤ grade 2. These were managed with diuretics. Common grade 3/4 toxicities were neutropenia (93.3 %), leukopenia (46.7 %) and anemia (33.3 %), and were managed with supportive therapy and/or FOLFIRI modification.

**Table 2:** Summary of treatment-emergent adverse events that occurred in 3 or more patients throughout the study (Safety analysis set)

MedDRA Preferred term	Treatment-emergent		Efatutazone-related	
	Overall	Grade 3 or higher	Overall	Grade 3 or higher
Hematotoxicity				
Neutropenia	15 (100.0)	14 (93.3)	7 (46.7)	6 (40.0)
Leukopenia	14 (93.3)	7 (46.7)	6 (40.0)	2 (13.3)
Anemia	13 (86.7)	5 (33.3)	11 (73.3)	4 (26.7)
Thrombocytopenia	10 (66.7)	1 (6.7)	3 (20.0)	1 (6.7)
Non-hematotoxicity				
Weight increase	15 (100.0)	0	15 (100.0)	0
Edema	12 (80.0)	1 (6.7)	10 (66.7)	1 (6.7)
Nausea	9 (60.0)	0	0	0
Vomiting	8 (53.3)	0	0	0
Alopecia	8 (53.3)	0	0	0
Fatigue	8 (53.3)	1 (6.7)	3 (20.0)	1 (6.7)
Decreased appetite	7 (46.7)	1 (6.7)	2 (13.3)	0
Diarrhea	7 (46.7)	0	1 (6.7)	0
Hypoalbuminemia	6 (40.0)	2 (13.3)	2 (13.3)	0
Constipation	5 (33.3)	0	3 (20.0)	0
Blood alkaline phosphatase increased	5 (33.3)	1 (6.7)	0	0
Blood creatinine increased	5 (33.3)	0	1 (6.7)	0
Hypercholesterolemia	4 (26.7)	1 (6.7)	3 (20.0)	0
Hyponatremia	4 (26.7)	1 (6.7)	4 (26.7)	1 (6.7)
Abdominal pain	4 (26.7)	1 (6.7)	2 (13.3)	0
Pyrexia	4 (26.7)	0	1 (6.7)	0
Gamma-glutamyltransferase increased	4 (26.7)	2 (13.3)	0	0
Nasopharyngitis	3 (20.0)	0	1 (6.7)	0
Stomatitis	3 (20.0)	0	1 (6.7)	0
Malaise	3 (20.0)	1 (6.7)	2 (13.3)	1 (6.7)
Aspartate aminotransferase increased	3 (20.0)	0	0	0

Values represent the number (%) of subjects

*MedDRA* Medical Dictionary for Regulatory Activities

A total of 14 (93.3 %) patients experienced at least 1 grade 3 or more severe TEAE: 3 (100 %) in the 0.25 BID group, and 11 (91.7 %) in the 0.50 mg BID groups. In the 0.25 mg BID group, 1 patient experienced grade 3 edema (44 days after the first administration of efatutazone), which was related to efatutazone and recovered with diuretics and temporary discontinuation of efatutazone. No patients experienced grade 3 or more severe weight increase. Five patients (including 2 patients with an UGT1A1 homozygous genotype) experienced grade 4 neutropenia (< 7 days duration), and 1 patient experienced grade 4 thrombocytopenia and grade 4 anemia.

No deaths were reported throughout the study period. Five patients (33.3 %) discontinued the study due to efatutazone-related TEAEs (fatigue, bronchitis, edema, anemia, and interstitial pneumonia). Three patients (20.0 %) experienced serious TEAEs related to both efatutazone and FOLFIRI, but no serious TEAEs during the DLT evaluation period. One patient on 0.25 mg BID had grade 3 fatigue and grade 2 bronchitis on Day 71. The study drugs were discontinued, and the patient received oxygen inhalation and antimicrobials, recovered of the fatigue and had relief of the bronchitis by Day 79. One patient on 0.50 mg BID had grade 3 interstitial pneumonia on Day 35. The study drugs were discontinued, steroid pulse therapy and antimicrobials were administered, and the patient experienced relief of the interstitial pneumonia by Day 55. One patient on 0.50 mg BID had grade 3 febrile neutropenia on Day 36, and grade 4 anemia and grade 4 neutropenia on Day 37. The patient received platelet transfusion, antimicrobials and granulocyte colony-stimulating factor for the anemia. Fever improved by Day 40 and anemia and neutropenia by Day 43.

### Efficacy

**Fig. 1:**
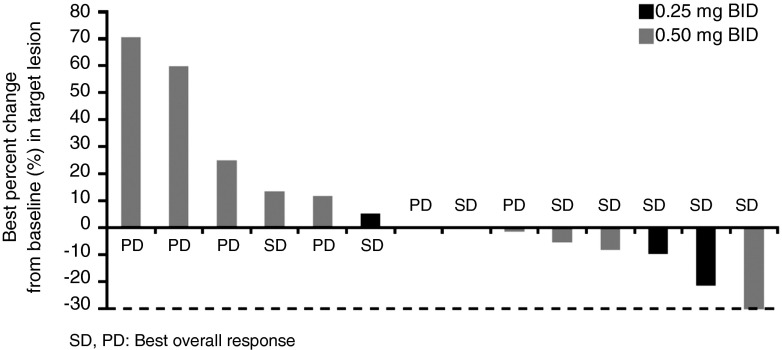
Waterfall plot of the best percent changes from baseline in the target lesion. Best percent change from baseline (%) in the target lesion = ([the minimum sum of the longest diameters at all measurement time points − the sum of the longest diameters at baseline] / [the sum of the longest diameters at baseline]) × 100. *BID* twice daily, *PD* progressive disease, *SD* stable disease

A total of 14 patients were evaluable for efficacy analysis, with ORR of 0 %, and DCR of 57.1 % (95 % confidence interval [CI]: 28.9, 82.3). Five (45.5 %) of 11 patients on efatutazone 0.50 mg BID had stable disease (SD). A waterfall plot of the best percentage changes from baseline in the target lesion is shown in Fig. 1.

Median (95 % CI) PFS was 158.5 (158–159) days in the 0.25 mg BID group, 73.0 (65–235) days in the 0.50 mg BID group, and 85.0 (70–159) days in the overall study population.

### Pharmacokinetics

**Fig. 2:**
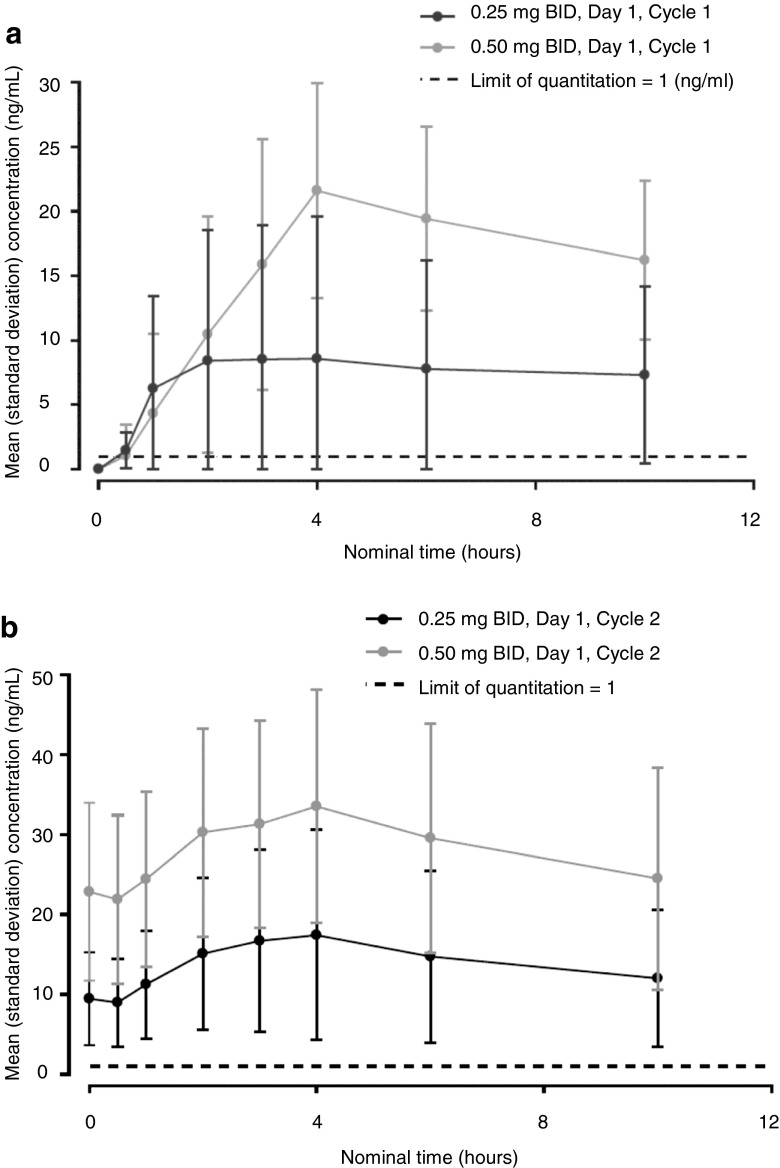
Concentration-time curve of the free form of efatutazone following oral dosing on Day 1 in Cycle 1 (single dose [a]) and Day 1 in Cycle 2 (repeated dose [b]). *BID* twice daily

Plasma concentration-time curves following administration on Day 1 (Cycles 1 and 2) are shown in Fig. 2. Although an absorption lag usually followed single-dose administration in the 0.50 mg cohort, the mean plasma concentration of efatutazone generally increased with repeated doses.

Pharmacokinetic values of the free form of efatutazone following oral administration on Day 1 (Cycles 1 and 2) are shown in Table 3. The C_max_ and AUC_tau_ in patients treated with efatutazone 0.50 mg were approximately 2 times those in patients treated with efatutazone 0.25 mg.

**Table 3:** Summary of pharmacokinetic parameters (Pharmacokinetic analysis set)

Parameter	0.25 mg BID		0.50 mg BID	
	Day 1 in Cycle 1^a^ (*n* = 3)	Day 1 in Cycle 2^b^ (*n* = 3)	Day 1 in Cycle 1^a^ (*n* = 12)	Day 1 in Cycle 2^b^ (*n* = 6)
C_max_ (ng/ml)	10.1 (9.5)	18.3 (13.2)	22.2 (7.97)	35.5 (14.7)
T_max_ (hours)	4.03 (2.00–8.03)	3.25 (1.98–3.95)	4.08 (1.97–6.00)	2.99 (1.97–4.03)
AUC_last_ (ng∙h/ml)	60.9 (66.9)	125 (85.0)	127 (52.9)	255 (105)
AUC_tau_ (ng∙h/ml)	116 (99.3)	159 (110)	193 (66.5)	325 (154)
C_trough_ (ng/ml)	–	8.68 (5.91)	–	21.1 (10.8)

For C_max_, AUC_last,_ AUC_tau,_ and C_trough_, values represent the means (standard deviation)

For T_max_, values represent the median (range)

*AUC*_*last*_ area under the concentration-time curve from zero to the last quantifiable concentration, *AUC*_*tau*_ area under the concentration-time curve during the dosing interval, *BID* twice daily, *C*_*max*_ maximum plasma concentration, *C*_*trough*_ trough plasma concentration, *n* number of subjects, *T*_*max*_ time to reach the maximum plasma concentration

^a^Following single-dose administration

^b^Following repeated-dose administration

The trough plasma concentration did not increase with repeated doses.

### Biomarkers

**Fig. 3:**
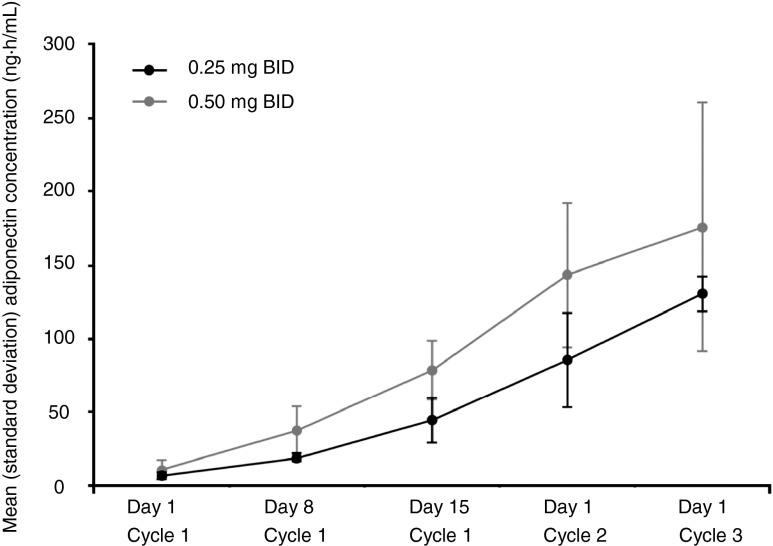
Time course of plasma adiponectin levels. *BID* twice daily

The plasma adiponectin levels increased over time and increased dose as shown in Fig. 3.

There were no apparent differences in the PPARγ expression levels in archived tumor specimens between patients with SD and those with PD (Mean [range] H scores in nucleus, 106.4 [5–180] versus 155.8 [5–240]; H score = ∑[the staining intensity × the occupied percentage of positive cells]). RXR expression levels were not significantly different in the nucleus H scores between patients with SD and those with PD (20.0 [0–70] versus 25.0 [0–75]).

## Discussion

This is the first study of efatutazone combined with FOLFIRI evaluating the safety profile and pharmacokinetics of efatutazone as second-line therapy in Japanese patients with mCRC. The safety profile in this study is consistent with that in previous Phase 1 studies (Ref. [12] and the poster presentation mentioned in the “Introduction”). Compared with the toxicity data of FOLFIRI as second-line chemotherapy for mCRC reported in a FIRIS (IRIS [irinotecan plus S-1] versus FOLFIRI) study [14], the toxicity profile of efatutazone plus FOLFIRI was almost identical to that of FOLFIRI alone, adverse effects including hematotoxicity were more frequent with efatutazone combined with FOLFIRI than with FOLFIRI alone. This higher incidences of adverse effects may partly be attributable to the initial irinotecan IV infusion dose (180 mg/m^2^) administered in this study, which is rather high for ethnic Japanese patients. In the FIRIS study, irinotecan 150 mg/m^2^ IV infusion was used [14]. Most adverse effects, including hematotoxicity, were manageable with supportive therapy and discontinuation/modification of efatutazone and/or FOLFIRI.

Preliminary efficacy results appeared to be comparable with those of second-line FOLFIRI therapy for advanced CRC (4 % PR, 30 % SD) reported in a randomized GERCOR study (randomized control study with FOLFIRI and FOLFOX) [13]. In a previous US Phase 1 study in 27 patients with advanced solid malignancies, 1 (3.7 %) patient with myxoid liposarcoma achieved sustained PR (690 days on treatment), and 10 (37.0 %) patients had SD for ≥ 60 days [12]. In a Japanese Phase 1 study in 13 patients with metastatic solid tumors, 1 (7.7 %) patient with thymic cancer achieved unconfirmed PR (> 210 days on treatment), and 3 (23.1 %) patients had SD (75–170 days on treatment) (the poster presentation mentioned in the “Introduction”). Based on the efficacy results of the present and previous Phase 1 studies, efatutazone is a potential clinically useful anticancer drug.

Efatutazone did not affect plasma concentrations of SN-38 (data not shown). Adiponectin is secreted by adipocytes in response to PPARγ agonist-induced gene expression in humans and rodents [15]. Since the plasma adiponectin level in colorectal adenoma patients is significantly lower than age-, sex-, and body mass index-matched non-cancer controls, it is considered a good biomarker for carcinogenesis and progression of colorectal adenoma [11]. Patients whose plasma adiponectin concentration reached ≥ 100 μg/mL tended to have longer PFS than patients with adiponectin < 100 μg/mL (mean [range] PFS, 106.9 [26–235] versus 60.6 [36–76] days). The clinical relevance of increased adiponectin levels and efatutazone treatment has to be studied further.

In a US Phase 1 study, archived tumor specimens of patients with SD or PR showed significantly higher PPARγ and RXR expression than those with PD [12]. In the present study, such differences could not be confirmed.

In conclusion, efatutazone at doses of 0.25 and 0.50 mg BID in combination with FOLFIRI demonstrates an acceptable safety profile and evidence of disease control in Japanese patients with mCRC. The RD in combination with FOLFIRI is 0.50 mg BID. The results of a randomized Phase 2 study of efatutazone in combination with FOLFIRI compared with FOLFIRI alone will also be reported soon.
